# Forensic Analysis of Head Traumas: Can Biomechanics Shed Light?—A Case Report

**DOI:** 10.3390/diagnostics16050766

**Published:** 2026-03-04

**Authors:** Carmen Rezek, Yves Godio-Raboutet, Maxime Llari, Lucile Tuchtan, Caroline Capuani, Catherine Boval, Marie-Dominique Piercecchi, Lionel Thollon, Clémence Delteil

**Affiliations:** 1 LBA, Aix Marseille University, University Gustave Eiffel, 13015 Marseille, France; carmen.rezek@etu.univ-amu.fr (C.R.); yves.godio-raboutet@univ-eiffel.fr (Y.G.-R.); maxime.llari@univ-eiffel.fr (M.L.); lionel.thollon@univ-eiffel.fr (L.T.); 2 CNRS, EFS, ADES, Aix Marseille University, 13015 Marseille, France; lucile.tuchtan@ap-hm.fr (L.T.); marie-dominique.piercecchi@ap-hm.fr (M.-D.P.); 3 Forensic Department, Assistance Publique-Hôpitaux de Marseille, La Timone, 264 rue St Pierre, 13005 Marseille, France; caroline.capuani@ap-hm.fr (C.C.); catherine.boval@ap-hm.fr (C.B.)

**Keywords:** finite element modeling, multibody modeling, traumatic brain injury, forensic investigation, pathology

## Abstract

**Background and Clinical Significance:** Traumatic brain injuries (TBI), most frequently caused by falls, represent a major source of morbidity and mortality and pose significant challenges in forensic investigations, especially when events are unwitnessed or testimonies conflict. Despite advances in imaging and autopsy, reconstructing the mechanism of head trauma often remains impossible. The objective of this study is to assess how biomechanical modeling can support forensic practitioners by narrowing the range of plausible scenarios and strengthening evidence-based interpretation in complex medico-legal contexts, without seeking to establish legal causality or certainty. **Case Presentation:** This case report investigates forensic biomechanics as a decision-support tool using a combined multibody and finite element (FE) modeling approach. An initial set of twenty-five scenarios, derived from witness statements and investigative data, was reconstructed to simulate potential fall- and assault-related mechanisms. Multibody simulations with the human facet model were first performed to estimate head impact velocities and orientations. These parameters were then applied to an FE head model to evaluate tissue response. **Conclusions:** Skull fracture patterns and intracerebral von Mises stress distributions were analyzed and systematically compared with clinical, radiological, and autopsy findings. Although simulated stress magnitudes were generally lower than injury thresholds reported in the literature, several scenarios reproduced fracture propagation and intracerebral stress patterns consistent with the documented lesions, including corpus callosum involvement. This multidisciplinary approach highlights the growing role of biomechanics in forensic investigations and forensic anthropology.

## 1. Introduction

Over the years, it has been shown that falls are the leading cause of hospitalization for traumatic brain injuries, accounting for 52% of cases [1]. Traumatic brain injuries stand out as the primary cause of disability and death among all other types of injury, imposing significant burdens on individuals, societies, and healthcare facilities [2,3]. Beyond their clinical impact, traumatic brain injuries represent a significant challenge in forensic practice, particularly when circumstances are unwitnessed or when testimonies are contradictory. In such situations, determining the precise trauma mechanism becomes essential for forensic interpretation.

Traumatic brain injuries encompass a wide spectrum of lesions, varying in severity and mechanism. They may present as focal injuries, such as contusions, skull fractures, and intracranial hemorrhages, or as diffuse injuries, including traumatic axonal injury resulting from rapid acceleration–deceleration or rotational forces [4,5]. Secondary lesions related to ischemia, edema, or systemic complications may further complicate interpretation. While their diagnosis is accessible through imaging or autopsy, understanding the mechanisms underlying the injuries is often not possible.

To investigate such injuries, numerical modeling has become an increasingly valuable tool in forensic biomechanics. Multibody models are mathematical representations used to simulate the dynamic behavior of interconnected rigid body segments within a given scenario. When applied to trauma reconstruction, such models allow evaluation of potential scenarios in light of witness statements and observed injury patterns through kinematic analysis. This initial modeling stage also provides an injury threshold (HIC15, VC, AIS-based injury probabilities), impact velocity, and rotational components, which are essential for understanding the underlying injury mechanisms. For example, Adamec et al. reconstructed two separate events involving a fall from a window and a fall into a deep shaft, helping to clarify different aspects of fall dynamics [6]. Hamel et al. investigated the effects of fall conditions and biological variability on skull fracture mechanisms resulting from falls [7], while Thollon et al. analyzed skull fractures following uncontrolled release during hanging [8]. Muggenthaler et al. reconstructed a balcony fall to clarify whether the incident was accidental or homicidal [9], and more recently, Erol and Karagöz applied multibody modeling to differentiate accidental, suicidal, and homicidal falls, illustrating the ongoing utility of numerical simulations in forensic scenarios [10].

In parallel, finite element (FE) modeling has been increasingly used to study cranial trauma. Initially developed for accident analysis and injury prevention, FE approaches are now recognized as useful tools in forensic investigations. Li et al. examined differences in skull fracture patterns between accidental injuries and suspected abuse cases [11]. Raul et al. used a head FE model developed at the University Louis Pasteur of Strasbourg to analyze a case involving two consecutive falls, distinguishing lesions caused by each fall [12]. Tuchtan et al. studied force transmission to the skull during mandibular impacts, demonstrating how localized impacts propagate stress throughout cranial structures [13]. Finally, Zhou et al. investigated pediatric cranial trauma using a FE model of a 10-year-old child, highlighting age-specific differences in injury mechanisms [14].

Although multi-body modeling and finite element modeling have been used for many years to reconstruct falls or differentiate accidental injuries from those resulting from assault, their combined use remains very limited in practice. However, these two approaches are complementary: multi-body modeling can be used to describe the overall kinematics of the body and dynamic interactions, while finite element modeling provides a detailed analysis of tissue stresses and injury mechanisms. The lack of systematic integration of these methods therefore constitutes a methodological shortcoming that may limit the accuracy of biomechanical reconstructions.

Interdisciplinary research has played a crucial role in the development of biomechanical techniques that improve forensic investigations, particularly in cases of unwitnessed and/or suspected homicide, helping forensic experts to determine whether the incident resulted from an accident or an assault [6,13,15,16,17].

The present study aims to assess whether an integrated multibody and FE modeling framework can serve as a decision-support tool in forensic investigations. Through this case study, we demonstrate how biomechanical modeling can function as a practical aid in real-world forensic practice. We hypothesize that by comparing simulated kinematic conditions and intracranial stress distributions with clinical, radiological, and autopsy findings, it is possible to systematically exclude biomechanically implausible scenarios. This process narrows the range of plausible mechanisms, without establishing legal causality.

## 2. Case Description

A 70–80-year-old man (body mass index: 23.6) was found unconscious on the sidewalk and subsequently admitted to the intensive care unit. Upon examination, he was found to have a contused wound on the right occipital region, measuring 5 cm by 2 cm, with irregular edges. The patient’s CT scan revealed a fracture of the right occipital bone extending to the right occipital condyle, a non-displaced fracture of the apex of the right petrous bone, acute subdural hematoma over the left and right cerebral hemispheres and the tentorium cerebelli, and bilateral frontotemporal post-traumatic subarachnoid hemorrhagic lesions.

The CT scans were obtained and recorded in Digital Imaging and Communications in Medicine (DICOM) format. Imaging was performed using a Definition^®^ CT scanner (Siemens, Erlangen, Germany) with 64 rows of detectors. The crania-facial complex was scanned, and images were obtained without intravenous administration of iodinated contrast material. Images were obtained in 0.5 mm contiguous sections. These images were then processed in one of two ways: three-dimensional (3D) reconstructions were obtained on the CT workstation and then transferred via a picture archiving and communication system (PACS), or the images were transferred via CD-ROM and 3D reconstructions were carried out using the software of a dedicated workstation (SECTRA^®^, Sectra AB Core, Linköping, Sweden). The CT data were reformatted to depict fracture in the various anatomical planes (coronal, sagittal, transverse), based first on the multiplanar sections and then on the 3D reconstructions. An experienced forensic radiologist analyzed the images.

**Figure 1:**
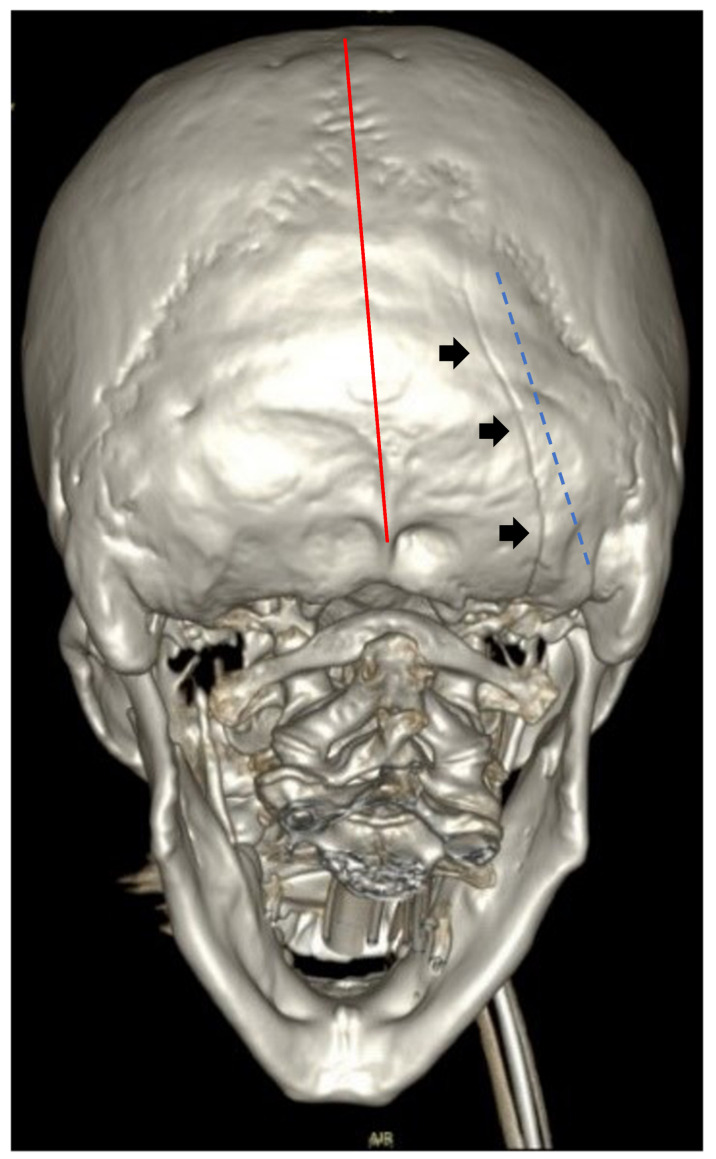
Three-dimensional reconstruction of the patient’s fracture line (dark arrows) based on CT images. Red line corresponds to the sagittal plane; blue dotted line highlights the oblique orientation of the fracture.

The 3D reconstruction of the fracture line in the patient based on CT images is represented in Figure 1.

**Figure 2:**
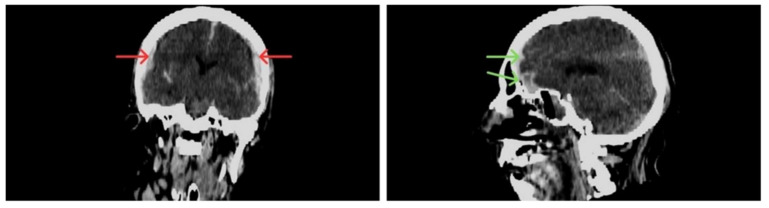
Post-impact CT scans of the subject’s head taken in the emergency department upon admission. The subdural hematoma is indicated with the red arrows and the frontal subarachnoid hemorrhage is marked with the green arrows.

**Figure 3:**
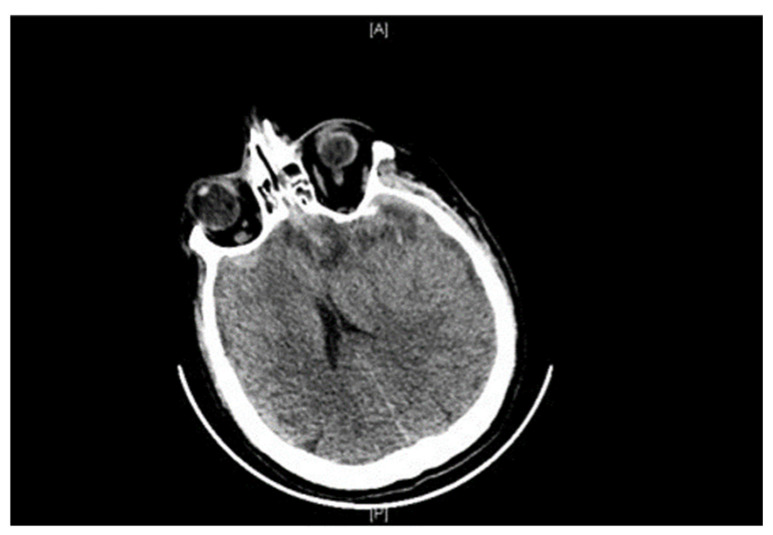
Ante-mortem follow-up CT-scan.

The abundance of subarachnoid hemorrhage did not formally identify a contusion (Figure 2). However, on a follow-up CT scan, petechiae were seen in the bilateral temporal region, extending into the left retro-orbital region, with bilateral frontal cortico-subcortical hypodensity with edema (Figure 3). These lesions are compatible with contusions (not seen at the initial stage) or secondary lesions related to ischemia.

As the police had only been informed by medical staff through a report to the prosecutor, no investigation of the crime scene could be carried out and no usable video surveillance was available. The only evidence that could be gathered came from the testimonies of those present, some of whom were visible on cameras located some distance from the scene of the alleged assault.

Few days later, after the victim’s death, forensic examination revealed a contusion of the right temporal region, characterized by a blackish ecchymosis of the right lower eyelid extending to the right temporal region and a retro-auricular violaceous ecchymosis, as well as two quasi-healed wounds at the level of the right superciliary arch and in the right occipital region.

Autopsy and neuropathological examination revealed lesions including a right paramedian occipital fracture involving the occipital condyle, transfixing over 8 cm and spreading internally only to the right parietal region over 5 cm a bilateral frontal subarachnoid hemorrhage with a 1 cm left inferior frontal contusion, a 1 cm anterior left temporal contusion and a median, peduncular hematoma, measuring 2 × 1 cm. Following the immunostaining anti-beta amyloid precursor, axonal damage was observed in the white matter including in the corpus callosum and next to the contusions.

In the light of divergent witness testimonies, one party claimed that the patient fell from his height, while others suggested he received a punch. The intensive care physician suggested a fall from the man’s height. However, the forensic pathologist identified two contusive mechanisms, suggesting two blunt force impacts on the right temporal and right occipital regions.

## 3. Material and Methods

The study design as a retrospective medico-legal case analysis integrating two main steps: (1) the reconstruction of possible fall scenarios using multibody modeling and (2) the analysis of head injuries using FE modeling. The research was carried out at the Laboratory of Applied Biomechanics in Marseille.

### 3.1. Step 1: Reconstruction of Fall Scenarios Using Multibody Modeling

The multibody model, a mathematical representation for simulating the dynamic behavior of interconnected rigid bodies, was essential in the initial stage of our study. This model was incorporated into Madymo Software (V. 2020.1, Siemens Industry Software and Services BV, The Hague, The Netherlands), an advanced automotive safety simulation software that integrates multibody dynamics.

The human facet model (HFM) was used with the anthropometric measurements available for our clinical subject via autopsy data. The HFM was initially developed, validated, and released by Madymo Software to investigate vehicle occupant safety [18]. In 2023, it was validated against experimental blunt impacts to the back [19].

A hundred simulations were performed to evaluate the possible scenarios and identify the initial impact conditions.

Among these 100 simulated configurations, 75 were excluded during the preliminary selection phase. Exclusion was based on the following criteria: (1) body kinematics considered unrealistic or incompatible with a plausible fall sequence; (2) head impacts occurring on anatomical regions inconsistent with the documented injuries.

The remaining 25 scenarios, judged both biomechanically plausible and consistent with the injury assessment, were retained for detailed analysis.

The precise point of impact, whether the head struck the curb of the sidewalk or the ground, was not specified in the forensic report. Therefore, for each angle of impact between the punch and the face, equivalent scenarios were performed, with simulations conducted both on the ground and on an 18 cm-high curb.

Twenty-five scenarios out of the one hundred already indicated were selected based on the most logical interpretations of the testimonies. Among these, two scenarios involved the patient falling from his height without any external force, two scenarios entailed the patient being struck from behind, impacting his right occipital zone with a punch (7 × 10 cm), and two additional scenarios involved a similar mechanism but with the impact occurring from a (3 × 10 cm, 2 kg) iron bar.

The remaining simulations involved the patient being punched in the face, impacting the region below his right eyelid. The punch (7 × 10 cm) had a mass of 2.85 kg and a velocity of 7.5 m/s. The punch’s mass, force, and velocity selection was based on a study by Adamec et al. [20].

**Figure 4:**
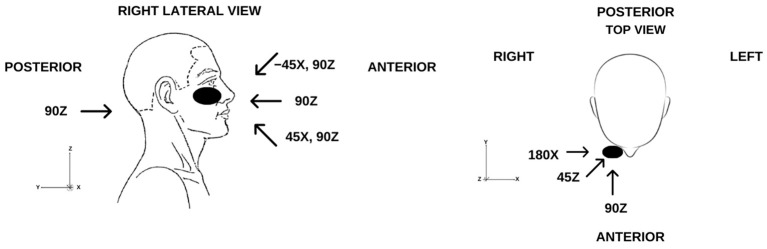
Illustration of the different punch impact angles.

The different angles of impact are illustrated in Figure 4.

**Figure 5:**
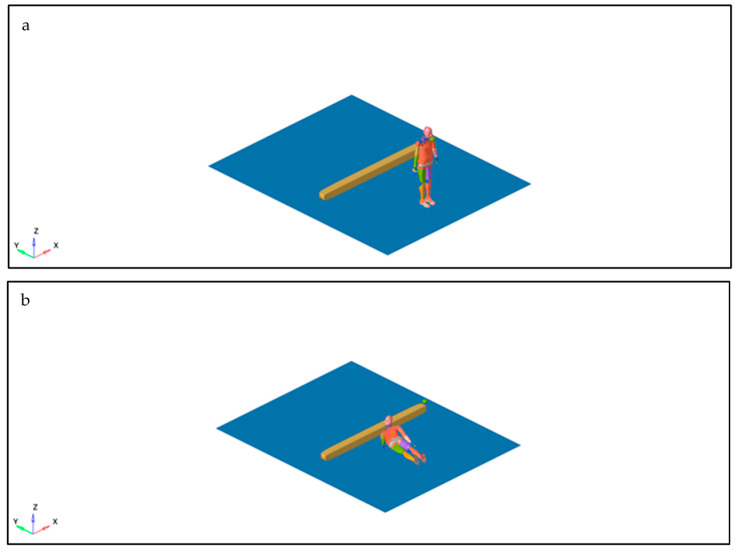
Capture of the multibody model at the moment of the punch (**a**); Capture of the multibody model impacting the sidewalk (**b**).

An example of a simulation impacting the multibody model with an angle of 90 degrees along the *Z*-axis is illustrated in Figure 5a,b.

The initial selection of scenarios prioritized the impact zone, focusing on whether it corresponded to the area of interest present in the patient. In succession, the angles and velocities of impact between the head and the ground or the head and the curb were extracted for the selected scenarios and applied as input data in the FE modeling.

### 3.2. Step 2: Analysis of Head Injuries Using Finite Element Modeling

The FE Method represented the second step of our work, a numerical modeling technique used for studying the stresses (such as von Mises Stress) and strains of the studied model. Its use extends to various fields such as engineering, structural design, mechanics, fluid dynamics, electromagnetism, and even healthcare. This technique is particularly applied in biomechanics for predicting injuries and understanding injury mechanisms [21].

**Figure 6:**
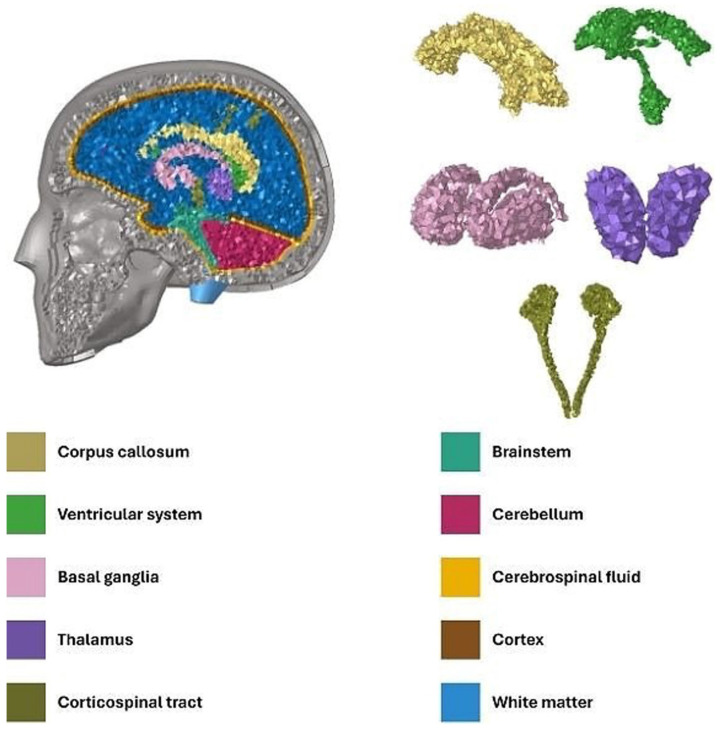
Left: Sagittal section of the FE human head model illustrating the intracerebral structures, with distinct anatomical components color-coded. Right: Isolated internal structures are displayed, including the corpus callosum (yellow), ventricular system (green), basal ganglia (pink), thalamus (violet), and corticospinal tract (khaki).

In this study, the head model, initially developed using 1 mm tomography developed by the Laboratory of Applied Biomechanics in Marseille, underwent multiple updates over time, incorporating new brain structures [7,13,16,22] (Figure 6).

Furthermore, this model was validated according to the experimental tests conducted by Nahum et al. in 1997 [23] that aimed to analyze the intracranial pressure during an impact to the head, by hitting the frontal region of the PMHS at different speeds using a rigid cylinder. Additionally, it was further evaluated using the experimental methodology developed by Hardy et al. in 2001 [24], which applied controlled accelerations to assess the displacements of specific brain regions.

The initial step involved verifying the compatibility of the FE head model with the patient’s skull and brain dimensions. This was achieved by 3D segmenting the patient’s brain using 3D Slicer 5.4.0 [25], with the segmentation based on the patient’s scan data, followed by taking various cerebral and osseous measurements from both skulls and brains. Then, the total volume of both models was calculated to determine the volume difference between the two.

Based on the impact angles obtained from the multibody modeling, the FE head model was positioned relative to the two models representing the curb of the sidewalk and the ground, both modeled as 2D surface elements, using HyperMesh 2021.2 software (Altair Engineering Inc., Detroit, MI, USA). Contact interfaces were established between the model and either the ground or the curb, with both surfaces (ground and curb) treated as rigid bodies during the simulations.

As a next step, the impact velocities extracted from multibody modeling were applied across different axes (x, y, z) using HyperCrash 2021.2 software (Altair Engineering Inc., Detroit, MI, USA). The simulations were processed using Radioss^®^ software (version 2021.2) (Altair Engineering Inc., Detroit, MI, USA) and analyzed using HyperView 2021.2 software (Altair Engineering Inc., Detroit, MI, USA). The analysis in HyperView included assessing the distribution of stresses and strains on the skull and the brain, as well as comparing the resulting injury patterns from simulations with those observed in the medical imaging of the patient, allowing the selection of the most probable scenarios. Von Mises stress was selected as the primary injury metric, as it has been shown to be particularly relevant for head impacts involving short-duration, high-rate loading conditions, such as punches or falls, and has been widely used in forensic biomechanical reconstructions.

## 4. Results

### 4.1. Multibody Modeling

The values of the punch’s mass and velocity were confirmed by evaluating the punch force, which was found in an acceptable range compared to the results of Adamec et al.’s study [20]. The obtained force values are illustrated in Table 1.

This approach allowed the chosen parameters to reproduce the experimental biomechanical conditions.

The first selection of the scenarios was mainly based on the location of impact on the patient’s head. If the impact zone of the scenario concerned our areas of interest, more precisely the right occipital zone when the patient was hit in the face, and the right temporal zone when the impact occurred in the back, this scenario was selected. Table 2 outlines the twelve selected scenarios.

In particular, in cases where the patient was struck at a 180-degree angle along the *X*-axis (as illustrated in Figure 4), the right occipital region has still not been affected, regardless of whether there has been contact with the ground or the sidewalk.

Two of the chosen scenarios, where the patient fell without any initial impact, were based on two possibilities. The first possibility is that the injuries to the right occipital and temporal regions were both caused by the fall, either onto the ground or the sidewalk. The second possibility is that the patient was first struck in the temporal region during a separate event and then fell from their height, sustaining additional injuries upon hitting the ground or sidewalk.

### 4.2. Finite Element Modeling

#### 4.2.1. Calculation of the Volume

The total volume between the patient’s 3D brain model and the FE Head model was compared to assess their correspondence. Table 3 presents the volume results, revealing a variation of 6.02% between the two models.

Regarding the FE modeling results, out of the twelve simulations already selected following the multibody modeling, six simulations were excluded as they did not match the clinical lesion profile: simulations 5 and 6 had an impact on the left occipital region; simulations 9 and 10 showed no impact on the right temporal region; and simulations 11 and 12 had no impact on the temporal region below the right eyelid but rather on the frontal region.

Accordingly, simulations 1, 2, 3, 4, 7, and 8 were selected for further analysis.

On the skull level, we observed various fracture profiles. Simulations 2, 3, and 4 presented a star fracture propagation profile, while simulations 1 and 7 displayed a circular propagation profile.

**Figure 7:**
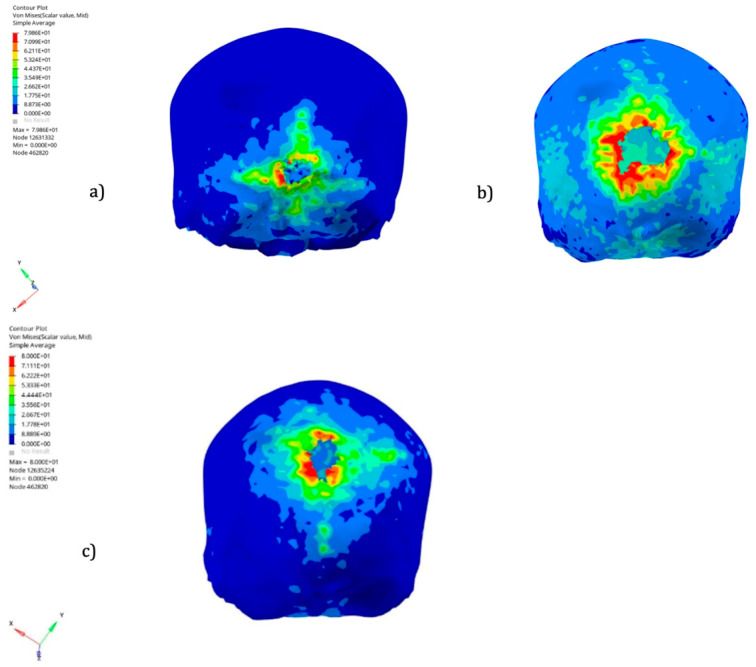
Skull fracture profiles. (**a**) stellate (**b**) Circular aspect (**c**) Towards the right and downward.

Simulation 8 showed a fracture propagation towards the right and downward, noting that this pattern most closely matches the clinical case (Figure 7).

The transmission of the impact occurs from posterior to anterior (occipital to frontal lobes) with preferential areas at the interhemispheric region, located on either side of the falx cerebri.

There is systematic involvement of the brainstem and cerebellum, with values that do not exceed the injury risk threshold, with propagation to the cerebral hemispheres.

During the initial milliseconds following the impact, the cerebellum, thalamus, basal ganglia, and corticospinal tract present minimal von Mises Stress levels.

On a cerebral level, the corpus callosum is affected by a stress threshold ranging from 3.30 to 12.2 kPa across all the simulations studied.

**Figure 8:**
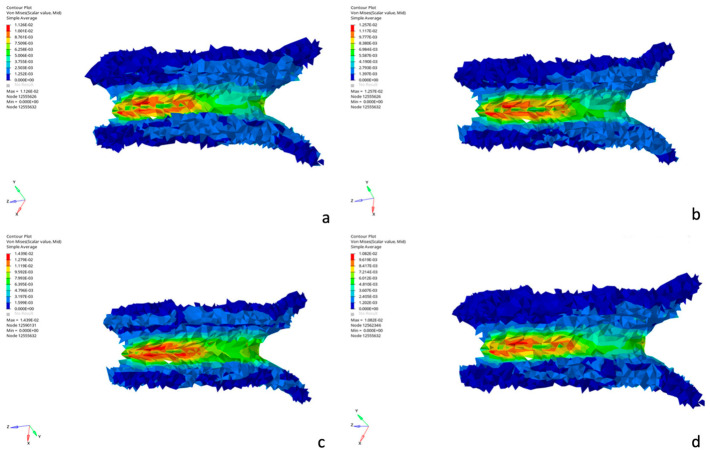
Von Mises Stress distribution in the corpus callosum in simulations 1, 2, 4, and 8. (**a**) Simulation 1 (**b**) Simulation 2 (**c**) Simulation 4 (**d**) Simulation 8.

The results showed an exceeded lesion threshold of the corpus callosum with values ranging from 9.6 to 12.2 kPa, except for simulations 3 and 7, which have values of 7.8 and 3.3 kPa, respectively, compared to Kleiven’s study, which indicates a lesion threshold of 8.4 kPa for the corpus callosum. These results indicate the presence of an axonal injury in simulations 1, 2, 4, and 8 [26], which shows coherence with our clinical case (Figure 8).

The macroscopic areas of contusion: left temporal and orbital regions show von Mises stress levels ranging from 0.5 to 4.7 kPa across the six selected simulations. We can note that the cortex and the white matter in simulation 4 and the white matter in simulation 8 were not affected in these regions.

**Figure 9:**
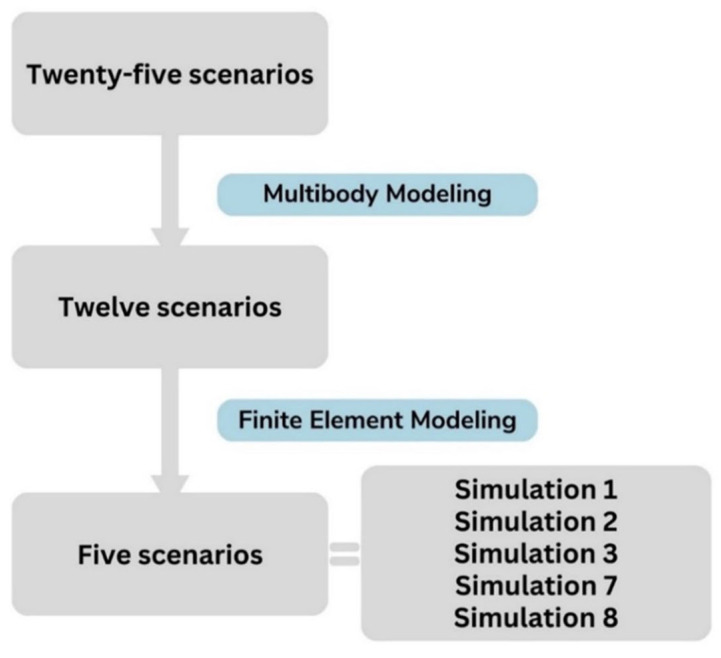
Flowchart illustrating the final selected scenarios.

Since the cerebellum and white matter were not affected in the cortico-subcortical regions in simulation 4, it can be excluded as it does not correspond to the patient’s clinical profile. Thus, we reduce the plausible scenarios from six to five simulations (Table 4, Figure 9).

Among the 100 simulated configurations, 25 scenarios were retained based on testimonies, 12 after kinematic consistency, 6 after FE injury comparison, and finally 5 plausible scenarios consistent with the clinical profile.

#### 4.2.2. Influence of the Impact Surface on Stress Propagation

There is a significant variation in von Mises stress thresholds between head impacts on the sidewalk (simulations 1, 3, and 7) and impacts on the ground (simulations 2, 4, and 8).

Concerning the cerebellum, the variation percentage is 14.28% between simulations 1 and 2, 54.16% between simulations 3 and 4, and 7.69% between simulations 7 and 8.

For the cortex, the variation percentage is 10.6% between simulations 1 and 2, and 41.37% between simulations 7 and 8.

Regarding the white matter, the variation percentage is 33.33% between simulations 1 and 2 and 80.76% between simulations 3 and 4.

These percentages indicate a higher von Mises stress threshold with simulations of the head impacting the ground than those of the head hitting the sidewalk.

The retained simulations indicate that the injury profile is most consistent with a two-step traumatic sequence involving an initial facial punch followed by a secondary occipital impact against the ground or curb. The initial punch likely modified the subject’s head velocity and body orientation prior to ground contact, influencing the direction and intensity of the occipital impact. FE analysis demonstrates that this secondary impact produced stress patterns compatible with the paramedian occipital fracture involving the occipital condyle.

## 5. Discussion

As we have seen, this case presents several forensic issues. On one hand, multiple versions of the trauma have been reported to law enforcement. Additionally, physicians have proposed several theories. The imaging performed at different times, as well as autopsy, allows the identification of injuries without determining whether they are primary or secondary cerebral lesions. However, it does enable the establishment of a reliable fracture profile. Forensic examinations attributed the cause of death to head trauma. However, only biomechanics allowed for an approximation of possible traumatic events.

Our methodology allowed us to narrow down twenty-five initial scenarios to five biomechanically plausible ones, underscoring the complexity of elucidating brain injury mechanisms. The selection process is detailed in a dedicated paragraph, which outlines the progressive exclusion criteria—including kinematic filtering, fracture pattern comparison, and intracerebral stress analysis—and discusses the forensic implications of this systematic approach.

In the upcoming discussion, we will analyze the implications of our findings and propose future research directions to better understand the underlying mechanisms responsible for brain injuries.

### 5.1. Multibody Modeling

The first step of the study involved conducting purely numerical simulations to reconstruct possible scenarios of falls. Several studies have used multibody modeling to identify injury thresholds [9,10]; in contrast, our approach also employs it to generate output data that serve as input for finite element simulations, enabling the analysis of more localized lesions. It would be beneficial to complement these with experimental tests on post-mortem human subjects (or crash test dummies) to validate the kinematics and impact forces used in the numerical simulations [27]. However, since the multibody model used has been validated in previous research on similar fall mechanisms [19], it may not be necessary to replicate all numerical simulations experimentally. Instead, it would be more prudent to select two or three numerically tested situations and compare them with the results obtained from experimental tests [20].

### 5.2. Finite Element Modeling

The second phase of the work used the head model developed at the Laboratory of Applied Biomechanics (LBA) to evaluate injury stresses. To ensure compatibility between the LBA FE head model and the clinical case skull, we compared their dimensions and calculated total brain volumes. Despite some discrepancies in skull measurements, brain volume proved crucial for determining impact responses, regardless of skull shape, supporting the use of the LBA FE head model [28].

Additionally, the properties of biological tissues can vary significantly between individuals (inter-individual variability) and even within the same individual (intra-individual variability). Furthermore, there are various skull morphologies, and within the same skull, thickness can vary depending on the areas studied. Based on the study of Rowbotham et al., the thickness determines the risk of differential fractures during blunt impacts [29]. The experimental data used to determine these characteristics may include measurement uncertainties, and individual differences might not be fully accounted for in the models [30]. A sensitivity study varying these properties could be conducted to assess their influence on the injury profile.

Moreover, brains present significant inter-individual variability in terms of size, shape, and mechanical properties [30]. It has also been suggested that women may have lower biomechanical thresholds, increasing their susceptibility to concussions [31].

FE models are often based on average or generic brains, typically using that of a male representing the 50th percentile, which may not accurately reflect real individual variability [32]. Therefore, it is necessary to personalize the models according to age, sex, and biomechanical properties of each individual’s biological tissues. While these generic models represent the “average” individuals within the studied categories, greater precision can be achieved through model personalization.

Biomechanical responses, such as intracranial pressure, stress, and strain within brain tissue, have been widely used in the assessment of brain injuries due to their strong correlations with injury mechanisms [33]. For head impacts occurring at velocities similar to those of a punch, von Mises stresses are particularly suitable for defining intracranial injuries [34].

A higher stress threshold was observed in simulations of head impacts against the ground compared to those against the sidewalk. This finding cannot be directly compared with existing literature due to differences in the FE model, highlighting how biomechanical properties, initial conditions, and model positioning can influence the results [7].

On a cerebral level, the von Mises stress levels in the macroscopic areas of contusion: left temporal and orbital regions are below the stress thresholds of a mild traumatic brain injury, found in the literature: 16.8 kPa according to Sahoo et al.’s study [35] and 18 kPa according to Willinger and Baumgartner [36], and even below the von Mises stress thresholds of a severe traumatic brain injury according to Willinger and Baumgartner [36] with a value of 38 kPa, suggesting that the observed lesions are probably resulting from secondary or tertiary processes rather than from the initial impact and its backlash, supporting the theory already indicated in the patient’s clinical profile. Therefore, establishing inclusion criteria for prognostication should be incorporated to study these possibilities [37], especially since secondary and tertiary injuries are still rarely studied in numerical simulations of severe head trauma.

In addition to this proposition, it is important to note that the multibody modeling accounted for the complete sequence, from the initial impact at the temporal region to the occipital impact on the ground. However, the FE modeling only considered the injuries resulting from the ground impact and did not account for the cumulative injuries from both sequences. Our methodology suggests that the observed occipital fracture resulted from a fall induced by an initial facial punch rather than a single occipital impact. By linking head kinematics from multibody simulations to stress patterns in the FE model, we provide a plausible explanation for the sequential trauma, which may not be apparent from imaging or autopsy alone. Using the punch impact on the head allowed us to obtain the head impact velocities on the ground or sidewalk, without being able to analyze the specific stresses from the punch itself. This finding underlines the importance of studying the complete traumatic sequence by combining both traumatic mechanisms and analyzing the resulting stresses, particularly in cases with conflicting testimonies [12]. Future work should aim to simulate the entire traumatic sequence within a single integrated FE framework in order to directly evaluate cumulative injury mechanisms and overlapping stress propagation generated by sequential impacts.

It is crucial to maintain objectivity and inclusivity when using this approach, ensuring that all possible scenarios are considered during testing. Following this principle, we narrowed down the initial twenty-five scenarios to the five most plausible ones that could explain the patient’s injuries. This selection was guided by the clinical information available, including the nature of the brain lesions and the specific locations of the injuries across different regions of the head, which made it possible to refine and narrow down the number of scenarios tested. This biomechanical approach does not aim to establish legal causality or certainty but rather to assist forensic experts in narrowing down biomechanically plausible scenarios consistent with the available clinical and autopsy findings.

Indeed, such an approach would provide a better understanding of the factors contributing to the observed lesions. Multidisciplinary approaches are an undeniable asset when it comes to solving these problems [38].

### 5.3. Practical Implications

Biomechanical modeling reduced 25 fall scenarios to 5 plausible outcomes.

Multibody and FE models enabled precise reconstruction of traumatic brain injury mechanisms.

Fracture morphology comparison can support impact localization.

Intracerebral stress distribution may help interpret deep structure involvement (e.g., corpus callosum).

Biomechanical modeling can assist forensic experts when testimonies conflict.

### 5.4. Limitations

The inherent limitations of a single case report.

Use of a generic FE head model rather than a fully patient-specific model.

Inter-individual variability in skull thickness and brain mechanical properties.

The inability to simulate cumulative sequential impacts within a single integrated FE sequence.

## 6. Conclusions

Our findings indicate that the observed brain injuries result from head impacts on the ground or sidewalk, with stress thresholds found to be lower than those indicated in the literature, possibly due to secondary or tertiary lesions. Personalized FE models, accounting for individual variability in biomechanical properties, and studying the entire traumatic sequence are crucial for determining injury mechanisms and outcomes.

This study helped us establish a solid methodology to assess injury scenarios, excluding unlikely scenarios and analyzing plausible ones. Biomechanical modeling does not establish certainty but assists forensic practitioners in narrowing plausible scenarios.

Biomechanics holds a promising potential in forensic anthropology, guiding practitioners by limiting the possible scenarios in light of the divergent testimonies. By integrating this multidisciplinary approach, researchers can enhance the understanding of the events surrounding deaths and traumas while contributing to the ongoing evolution of the forensics field.

In the near term, numerical simulations could be more frequently used alongside autopsies to support forensic analysis of cases.

## Figures and Tables

**Table 1:** Punch Force Comparison: Our study versus Adamec et al.’s. Force expressed in newtons (N) corresponds in International System base units to 1 N = 1 kg·m·s^−2^.

Punch		Force (N = 1 kg·m·s^−2^)
Literature (Adamec et al. [20])	Punch with the dominant hand	1665 ± 401
	Punch with the non-dominant hand	1506 ± 714
Simulation	Punch with an angle of impact of 45 degrees along the Z axis.	1317

**Table 2:** Selected multibody simulation scenarios.

	Impact 1 Location	Impact 1 Type	Impact 1 Orientation (°)	Impact 2 Surface	Head Contact Region at Impact 2	Head Velocity X at Impact (m/s)	Head Velocity Y at Impact (m/s)	Head Velocity Z at Impact (m/s)
1	Face	Punch	Z = 90°	Curb	Right occipital	0	1.5	−4.5
2	Face	Punch	Z = 90°	Ground	Occipital	−1.1	−1.5	−4.67
3	Face	Punch	Z = 45°	Curb	Occipital	0	2.9	−5.7
4	Face	Punch	Z = 45°	Ground	Occipital	0	0	−5.9
5	Face	Punch	X = −45°, Z = 90°	Curb	Right occipital	0	2.2	−3.7
6	Face	Punch	X = −45°, Z = 90°	Ground	Occipital	0	2.3	−4.1
7	Face	Punch	X = 45°, Z = 90°	Curb	Occipital	0	2.1	−3
8	Face	Punch	X = 45°, Z = 90°	Ground	Occipital	0	2.2	−3.5
9	-	-	-	Curb	Occipital	0	0.9	−5.6
10	-	-	-	Ground	Occipital	−0.2	1	−5.9
11	Behind	Punch	Z = 90°	Ground	Face	1	0	−2.9
12	Behind	Iron bar	Z = 90°	Ground	Face	−0.3	1.1	−3

**Table 3:** Results of the brain volume of both the Patient’s 3D brain model and the FE Head Model.

Model	Total Volume (mm^3^)
Patient’s 3D Head Model	1.42 × 10^6^
FE Head Model	1.51 × 10^6^

**Table 4:** Results of the final selected scenarios.

	Impact 1 Location	Impact 1 Type	Impact 1 Orientation	Impact 2 Surface
1	Face	Punch	Z = 90°	Curb
2	Face	Punch	Z = 90°	Ground
3	Face	Punch	Z = 45°	Curb
7	Face	Punch	X = 45° Z = 90°	Curb
8	Face	Punch	X = 45° Z = 90°	Ground

## Data Availability

The original contributions presented in this study are included in the article. Further inquiries can be directed to the corresponding author.
